# Analysis of the Embodied Energy and CO_2_ Emissions of Ready-Mixed Concrete: A Case Study in Cuenca, Ecuador

**DOI:** 10.3390/ma15144896

**Published:** 2022-07-14

**Authors:** Karla Vázquez-Calle, Vanessa Guillén-Mena, Felipe Quesada-Molina

**Affiliations:** 1 GAD Municipal de Azogues, Solano y Matovelle, Azogues 030102, Ecuador; arqvazquezkarla24@hotmail.com; 2 Department of Energy Engineering, Faculty of Engineering of Bilbao, University of the Basque Country UPV/EHU, Ingeniero Torres Quevedo Plaza 1, 48013 Bilbao, Spain; 3 Engineering, Industry and Construction Unit, Catholic University of Cuenca, Av. De las Américas S-N y Humboldt, Cuenca 010105, Ecuador; felipe.quesada@ucacue.edu.ec

**Keywords:** carbon footprint, embodied energy, environmental impact, inventory, life-cycle assessment

## Abstract

Concrete is the most commonly construction material used worldwide. In contrast to other countries, Ecuador lacks studies that determine the environmental impact of the production of construction materials. This research presents a quantification of embodied energy and CO_2_ emissions associated with the concrete production, using as a case study a ready-mixed concrete plant in the city of Cuenca, Ecuador. The study was based on the Life Cycle Assessment methodology established by ISO 14040 and ISO 14044, and the 2006 Intergovernmental Panel of Experts on Climate Change (IPCC) Guidelines for National Greenhouse Gas Inventories. The production of ready-mixed concrete was considered for one year, with a “gate to gate” approach including the “transport of raw material” to the concrete plant and the subsequent “transport of final product” to the construction site. The results revealed that to produce 1 m^3^ of ready-mixed concrete, its production required 568.69 MJ of energy, accompanied by 42.83 kg CO_2_. Indirect transport generates the greatest environmental impact, especially the “transport of raw materials”, which represents approximately 80% of the embodied energy and 79% of CO_2_ emission.

## 1. Introduction

At the global level, the construction industry engages in activity that consumes the most natural resources [1,2,3]. It is attributed 60% of the extracted raw material and it is associated with 40% of primary energy consumption and 33% of CO_2_ emissions [4,5]. This situation results in a decrease in resources for future generations [6]. Hence, the need to transform the guidelines for production and consumption from open processes of waste generation to closed cycle processes that optimize resources and protect the environment [7,8].

Concrete is the most used construction material in the world [9,10], with a global use of approximately 25 gigatons per year [11] and after water, it is the most consumed product on the planet [12]. Even though per kilogram it may represent a reduced environmental impact compared to other materials such as steel, glass, timber and brick, the volume of concrete used around the world (estimated at over 10 billion cubic meters per year) makes the industry contribute significantly to global CO_2_ emissions and consumes large amounts of natural resources [13]. Concrete plants consume 1000 million tons of water, between 1500 to 2000 million tons of cement and 10,000 million tons of aggregates [14,15]. Although about 4000 million tons or Portland cement are manufactured each year, only about half of the cement is used for concrete, the rest is reserved for mortar, plaster and blocks [15]. Additionally, for each ton of cement used, 1.65 tons of limestone and 0.6 tons of clay are required [16]. Also a large amount of fossil fuels are required [14]. That is, within the production of concrete, cement consumes the most energy and releases the most carbon dioxide [11]. Consequentially, concrete is responsible for approximately 8% of CO_2_ emissions worldwide [10]. Most of these emissions are attributed to the production of cement [11], due to its clinkerization process based on furnaces with high temperatures (1350–1400 °C) [17].

According to the study by [18], it was estimated that in 2015, 2400 million cubic meters of ready-mixed concrete were produced in the world. China is responsible for more than half of that volume, followed by the United States and the European Union (11% and 9%, respectively). In this scenario, Iberoamerican countries represent 5.8%; however, in order to meet its construction needs, Latin America can significantly increase per capita concrete consumption.

In Ecuador, according to the 2015 Annual Survey of Buildings (Construction Permits) of the National Institute of Statistics and Censuses [19], it is evident that in the construction industry, the tendency is to use concrete as the main material. The construction of new buildings was projected with the use of this material in 79.6% in its foundations, 92.7% in its structure and 54.2% as a covering.

In this context, with the objective of providing local information that contributes to the generation of a national database of construction materials, which currently does not exist [20], the present research proposes to quantify the embodied energy and CO_2_ emissions to the environment of each stage of ready-mixed concrete production. It will allow decision makers to seek better ways to minimize their associated impacts. It is also expected that this document will strengthen current sustainable initiatives and encourage the development of new ones. It is important to point out that the novelty of the study consists in the field application Life Cycle Assessment in a specific locality, corresponding to a region that lacks databases for the Life Cycle Inventory, such as South America. Therefore, in addition to the above, this research will serve as a reference for other studies in the region.

### 1.1. Studies on the Concrete

Concrete is used due to its excellent characteristics, which include the following: good compression resistance, good fatigue behavior, excellent water resistance, good fire behavior and low maintenance cost [21]. Ready-mixed concrete is dosed into the same plant or in a mixing truck and transported to the construction site [14]. It consists of a mixture of cement with water (paste), fine and coarse aggregate, which can also contain additives that improve its properties in both fresh and hardened states [21,22]. The paste constitutes approximately 25% to 40% of the total volume of the concrete, while the aggregates make up between 60% to 75% [23].

Concrete is a material that has grown in popularity due to its wide application and its large impact on the environment [24]. The potential impacts generated during the production of concrete have been identified and quantified especially through the Life Cycle Assessment (LCA) method that is based on international standards [25,26]. The main impact categories identified in concrete studies with LCA include global warming (especially CO_2_ emissions) and consumption of energy resources. Additionally, it is common to find studies about the use of raw materials [27].

In terms of global warming, CO_2_ in addition to being emitted during the concrete manufacture, also comes largely from the production of cement (as one of its main components) and from the acquisition of aggregates and raw materials [21,28,29].

In relation to the consumption of energy resources, according to [30] 2600 MJ and 4000 MJ are required to produce 1 m^3^ of simple concrete and reinforced concrete, respectively. While for the production of 1 m^3^ of concrete with natural aggregates, 1570.42 MJ are required in Serbia [31]. A study in Ireland determined that for the production of 1 kg of a typical concrete requires 1.08 MJ [32].

Regarding the consumption of raw materials, it has been identified in the literature that the aim is to reduce the amount of clinker used in the manufacturing of cement and the replacement of part of the raw material by adding recycled materials [33,34] or wastes [35] in the concrete. A study carried out in Ecuador determined that the production of one ton of cement required 3191.95 MJ of energy, of which 91.4% corresponds to the production of clinker [36]. For clinker reduction, alternative non-Portland clinkers were developed. They are man-made mineral materials, which can be used as binder similarly to Ordinary Portland cement (OPC)-based clinker. Although the benefits in terms of reduction in energy requirement and CO_2_ emissions are considerable, its main limitation is that the raw material cost is significantly higher than that of OPC [13]. Other options under exploration as partial or total replacement of OPC are alkali-activated binders and supplementary cementitious materials [37]. Even study [38] addresses the reduction of the amount of binder without affecting the technical characteristics of the concrete. To replace part of the raw material, some researchers [39,40] have studied the use of recycled concrete aggregate. Also some materials such as waste from construction and demolition replace the both the fine fraction and the large fraction of the aggregate in the production of concrete, while marble sludge and cement kiln dust (CKD) could replace the fine fraction of the aggregate [35,40]. Other aggregate options for concrete can be identified in [37].

Since the aggregates come from the soil, novel methods such as the one proposed by [41] could be used to adequately evaluate the physical and mechanical characteristics of the materials belongings to a certain zone. In this way, it is achieved that the knowledge about the characteristics of the materials is representative of the region and that the data is reliable [41]. This could have a positive impact on the materials used in the production of concrete, as it is hoped solutions will be increasingly sustainable without affecting the material’s durability.

Other measures that are considered to improve the environmental performance of concrete are related to increasing the efficiency of the process and significantly reducing the use of non-renewable energy [42]. It is also important that the development of methodological tools that allow for the quantification and assessment of environmental impacts at each stage of a product’s life cycle [14]. The research of [13] proposes some actions to be taken into account in cement plants to increase efficiency by adopting technological advances such as the use of a dry process, modern pre-calciners, a new type of clinker cooling system and vertical roller mills. Additionally, we consider the use of alternative fuels such as biogas, used oils, wood waste, among others, and the use of industrial by-products and waste-derived materials such as agricultural wastes, ashes, iron, and steel slags to partially replace limestone and clay. However, in any of the options it is important to verify that the quality of the product is not affected. In the same study, the authors explore other current carbon dioxide reduction alternatives, such as the adoption of carbon capture, utilization, and storage technologies.

### 1.2. Life Cycle Assessment Framework

The Life Cycle Assessment consists of four phases: Definition of goal and scope, Life Cycle Inventory Analysis (LCI), Life Cycle Impact Assessment (LCIA) and Life Cycle Interpretation [43]. In the first phase, the system boundary must be defined, which can be:

(1) From “cradle to grave” when it includes all the inputs/outputs of the processes that participate throughout its lifecycle, extraction of raw material, processing of materials for the manufacture of components, use of the product, and recycling or final management;
(2) From “cradle to gate” when the scope of the system boundary is limited to inputs/outputs from the extraction of raw materials until the product is placed on the market (leaving the concrete plant);
(3) From “gate to gate” when considering only the inputs/outputs of the manufacturing process of the product [44]. In the second phase, data corresponding to the inputs and outputs are collected for all the processes of the product system. Inputs such as raw materials and energy are quantified, and the outputs as emissions (to air, water and soil), and the product obtained, are also considered. In the third phase, the data collected in the inventory of inputs and outputs are translated to indicators of potential environmental impacts. Finally, in the fourth phase, the results of the LCI and LCIA are interpreted according to the objectives and scope initially defined, concluding with an analysis of results, and the formulation of conclusions [25].

Of the LCA phases, with the exception of European countries and the United States, the greatest difficulties are found in the LCI phase due to lengthy data collection processes, since complete inventories are generally not available or are not reliable. Furthermore, using foreign databases can lead to errors due to the technology and power source used, if not used properly.

This methodology is widely used to determine the environmental impacts of construction materials and several current studies on concrete use it [45,46,47,48]. However, it has been identified that some countries have developed some additional tools or standards that contribute to the different stages of the life cycle; for example, in The United States, the Tool for Reduction and Assessment of Chemical and Other Environmental Impacts (TRACI) was developed, while in Europe the Standard UNE-EN 15804:2012 is used [46,49]. In both cases they provide characterization factors for life cycle impact assessment. Likewise, other environmental assessment methods such as MFA (Material flow account) or SFA (Substance Flow Analysis) have been identified. MFA can be considered as a method to create an inventory for an LCA. It serves as a tool for the measurement of and prediction of environmental pressures from the use of materials in an economy. Additionally, SFA may be considered as a sub-set of MFA that allows for identifying specific environmental problems [50].

### 1.3. The Concrete Industry in Ecuador

The energy demand in Ecuador has increased considerably in a decade (2009–2019), going from 69 million BOE (Barrel of Oil Equivalent) to 94 million BOE [51]. According to the historical trend, the transport sector is the largest demander of national energy with an average value of 37.9 million BOE, while the construction and other, residential and industrial sectors are credited with 9.5, 12.7 and 13.3 million BOE, respectively. [51]. With the increase in energy consumption, greenhouse gas emissions also show the same trend; in 10 years, they have increased by 19.3%.

The concrete industry has an impact on the indicated sectors since, for example, cement (concrete component that generates the greatest environmental impacts) presented a per capita consumption of 347 kg/hab (year 2019) [52], with Ecuador included in the group of countries with the highest consumption of cement per inhabitant [36]. The largest production of cement occurs in the coastal area of the country, contributing with 62.8% of the total volume. Other areas such as the north and the center-south contribute with 18.6% each one [47]. In the construction sector, concrete is the most used material in new buildings (89.6%), in extensions (8.5%) and reconstructions (1.9%) [53]. The city of Cuenca is among the three cities with the highest number of buildings to be built and therefore is also part of the group of cities that consumes the most ready-mixed concrete (10.2%), after Guayaquil (38.1%) and Quito (30.8%). The standard that establishes the specifications for the manufacture and delivery to the user of ready mixed concrete in a fresh and non-hardened state is the Ecuadorian Technical Standard NTE INEN 1855-1 [54]. In general terms, a study carried out in Ecuador [47] determined that conventional concretes with a resistance between 18–40 MPa, are the most used, representing 88%, while high compressive strength concrete (≥40 MPa) and low compressive strength (≤18 MPa) represent 6.5% and 5.5%, respectively. Of the first group, the most used concretes have a compressive strength of 28 MPa and 21 MPa, representing 22.5% and 20.1%, respectively, of national production. The first is used in the construction of structural elements such as columns, beams and building foundations, and the second is commonly used in slabs. Of the structures built in 2019, 86% were made of reinforced concrete, 11% metal and 3% other materials. This information shows that there is an important use of both cement and concrete in the country, so it is necessary to look for more sustainable alternatives in their application. Even the use of aggregates is worrying because it is considered a non-renewable resource and in the country its extraction is increasing every year [55]. The research by [45] identified that there are few studies of sustainable concrete in South America. For example, they did not identify studies that use recycled aggregate to decrease virgin aggregates. In one of the main local concrete plants, it was found that the use of recycled aggregate in the production of concrete barely reaches 1% [56]. Therefore, in the country and in the region, there is a high potential for reducing embodied energy, CO_2_ emissions and virgin aggregates.

## 2. Materials and Methods

This study presents a quantitative, non-experimental, longitudinal approach carried out during a calendar year (2015). The main objective is to provide local data on environmental performance in the production of the ready-mixed concrete in two impact categories, corresponding to the consumption of energy resources and global warming (CO_2_ emissions). The research considers as a case study a plant located in the city of Cuenca, Ecuador under a “confidentiality agreement”.

The quantification of the data followed the Life Cycle Assessment methodology according to the principles established in the standards ISO 14040 and ISO 14044 [25,26]. Additionally, to quantify CO_2_ emissions, Tier I of the “2006 IPCC Guidelines for National Greenhouse Gas Inventories” established by the Intergovernmental Panel on Climate Change (IPCC) was applied [57].

Following the LCA framework, three phases were developed: Definition of the goal and scope, LCI and LCIA. The fourth phase (Interpretation) was considered in the Section 4.

### 2.1. Definition of the Goal and Scope

The objective was to quantify the impacts on the most relevant categories of the ready-mixed concrete production: consumption of energy resources, and global warming (specifically CO_2_ emissions). The system boundary considered was “gate-to-gate”, including the previous stage corresponding to the “transport of raw material”, as well as the subsequent stage “transport of final product”. At the system boundary, only the elementary flows of the production chain of manufacturing were considered, leaving aside the energy consumptions from the administration.

**Figure 1:**
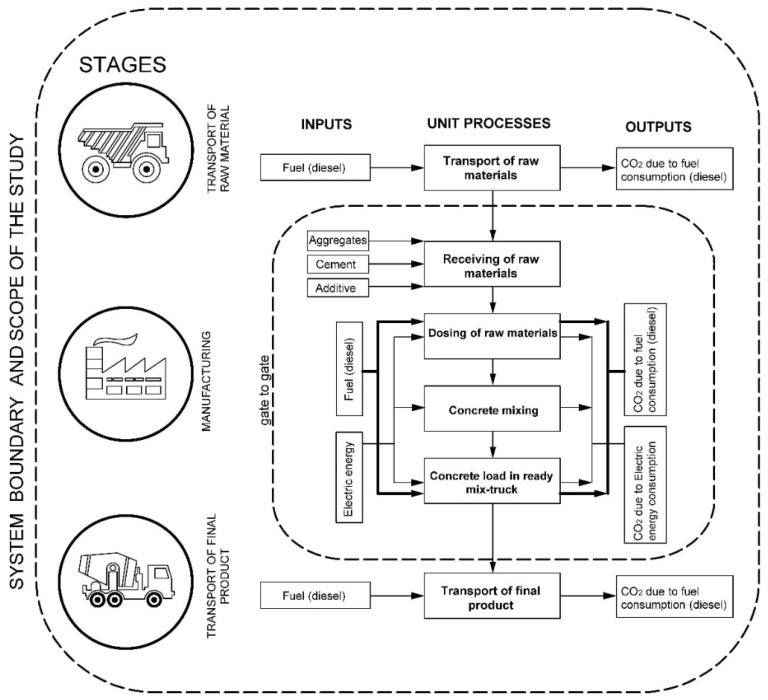
Flow chart of the ready-mixed concrete production.

Figure 1 presents the flow chart of the ready-mixed concrete production of the case study. It specifies input and output data of the system for each unit process: receiving and dosing of raw materials, mixing and concrete load of ready-mix trucks (within the boundaries of the plant). Additionally, the unit processes of the “transport of raw material” and “transport of final product” stages were considered (both outside plant boundaries). As input data, the raw material and the different types of energy consumed were considered, and as outputs, the product generated and the emissions emitted to the environment were taken into account.

The four processes involved in the production of ready mixed concrete within the plant are briefly described below:

(1) Receiving of raw materials: Mainly aggregates, cement and additives. Aggregates: Crushed stone with diameters of 38 and 19 mm and river sand. The product is weighed and stored in reinforced concrete compartments. Cement: It is transported in bulk from the main plant located in the city of Guayaquil. The trucks are weighed upon arrival and unload their product into cement storage silos. Additives: They are supplied wholesale by their supplier and arrive at the plant by means of cargo trucks. The most used additives are plasticizers, retardants, and accelerators.
(2) Dosing of raw materials: By means of a front-wheel loader, the different types of aggregates are taken to the reception hopper, the aggregates are supplied by means of a conveyor belt and placed in the compartments proper to each type. In the dosing hopper, there is a scale to dose by weight the mixture of aggregates for each batch of concrete to be manufactured. Once the cement is weighed, it is transported to the pre-mixer drum. The aggregate mix, still dry, is transported to the pre-mixer drum, where it will be mixed with the cement, water, and additives. The water that will be used is stored in a tank; it is pumped, and its dosage depends directly on the automatic dosage system. The additives are dosed and then, they are pumped to the premix tank. When all the dosing has been completed, mixing is carried out until the characteristics of the desired concrete are obtained.
(3) Concrete mixing: It is performed in the pre-mixing drum.
(4) Concrete load in ready-mix truck: Once mixed and homogenized, it is passed to the mixer through an automatic system from the dispatch area.

Once the final product has been delivered, the mixer truck returns to the plant for washing. This consists of sediment separation and water recycling. The aggregates return to the aggregate stock and the water to the raw-material-dosing system by means of a pump.

The functional unit chosen consisted in 1 m^3^ of ready-mixed concrete. In the impact category “consumption of energy resources”, the annual embodied energy was quantified in MJ/m^3^, and in the gases emitted to the atmosphere were expressed in kg CO_2_/m^3^ of the material manufactured [14,58,59].

### 2.2. Life Cycle Inventory (LCI)

The inputs and outputs of each unit processes specified in the flow chart of the ready mixed concrete production of the case studied were considered. The information was obtained from an analysis of the monthly and annual production databases of the plant.

For the quantification of fuel for transport of raw material, data provided by the suppliers through a survey of information gathering in the field was used.

#### 2.2.1. Inputs and Calculation

Raw materials: Each raw material supplied to the plant during a year was quantified. Coarse aggregate (3/4 gravel, 3/4 crushed, 3/8 crushed), fine aggregate (sand), cement and chemical additives.

Fuels: To quantify the fuel consumed in the first stage “transport of raw material”, information about the distance from the suppliers to the gate of the concrete plant, truck performance and return conditions (empty or loaded), was required. This study considered empty return trips. In the second stage “Manufacturing”, the fuel required in the operation of the front loader to transfer the aggregates to the receiving hopper, from which the dosing process start, was quantified. In addition, fuel was necessary in the ready mix-truck during the concrete loading. In the final stage “transport of final product”, the fuel consumed in the operation of the ready-mix truck to transport the concrete to the construction site was quantified. The trip back to the plant was also considered because the truck returns empty. To determine the embodied energy by transport, the conversion factor established by the SEAP Guidelines Part II was applied. The case study consumed, “diesel fuel”; therefore, the corresponding factor is 10 kWh/lt [60].

Electricity: It was only required in the second stage (manufacturing), during the dosing, mixing and concrete load processes.

#### 2.2.2. Outputs and Calculation

Final product: The total volume produced (m^3^) by the premix plant over one year was considered as a single type of concrete.

CO_2_ emissions (fuel and EE): To determine CO_2_ emissions derived from the use of fuel (diesel), Tier I established by 2006 IPCC Guidelines-Energy (Stationary Combustion) was applied, using Equation (1) [57]. The units of measurement established by SEAP Guidelines were used [60].

Emissions GHG, Fuel (gGEI) = Fuel Consumption Fuel (kWh) × Emission FactorGHG, Fuel (gGEI/kWh)(1)

where:

- Emissions GHG, Fuel: Emissions of a given GHG by type of fuel;
- Fuel Consumption Fuel: Amount of fuel combusted;
- Emission Factor GHG, Fuel: Default emission factor of a given GHG by type of fuel.

For CO_2_, it includes the carbon oxidation factor, assumed to be 1. The CO_2_ emission factor applied is 267 g CO_2_/kWh for diesel fuel [60].

To calculate the CO_2_ emissions from annual electricity consumption, the basic equation established in the 2006 IPCC Guidelines—General Guidance and Reporting [61] was applied:

Emissions = AD × EF(2)

where:

- AD: Activity data
- EF: Emission Factor

AD, it was considered the total electricity consumed during one year in the ready mixed concrete production. Additionally, for EF, the value of 0.6760 t CO_2_/MWh eq obtained from the National Interconnected System of Ecuador was applied [62].

### 2.3. Life Cycle Impact Assessment (LCIA)

This phase consists of transforming the data collected in the LCI into potential indicators of environmental impact. Two categories were considered: “Consumption of energy resources” and “Global warming” (CO_2_ emissions for being a representative GHG within the concrete production). The LCIA is developed in the Section 3.

## 3. Results

### 3.1. Input Data

#### 3.1.1. Raw Material Consumption

**Figure 2:**
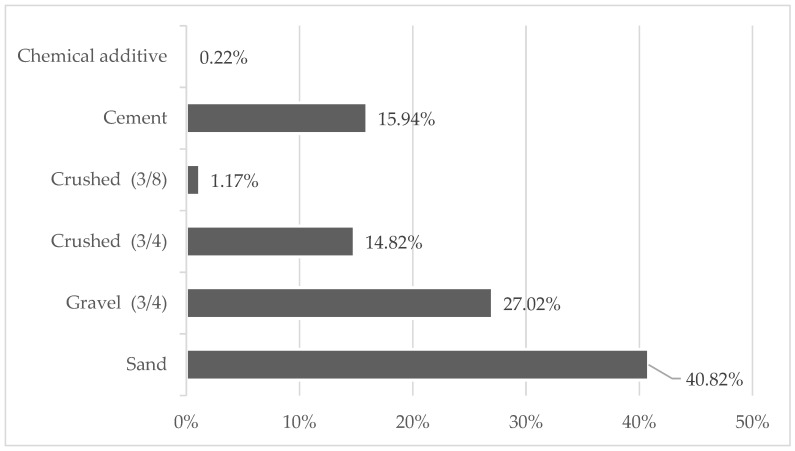
Quantity of each raw material entered during a year in relation to the total.

There are six raw materials that entered the plant during the year of study (without considering the water). The most consumed raw material was the sand (fine aggregate) represented by 40.82% and the least consumed was the chemical additive with 0.22%. Figure 2 reveals that aggregates, in general, were the most consumed, reaching 83.83% and the difference was mainly represented by cement with 15.94%. For quantification, the total mass in tons of each of the raw materials that entered the plant during the year of study was considered. These percentages presented in the case of Ecuador are similar to research carried out in Sweden and Ireland (without considering water). In these countries, the use of aggregates represents approximately 75% and 83.45%, while cement represents 15% and 16.6%, respectively, [50,63]. Compared to a national study, the data obtained are within the range defined by [47].

#### 3.1.2. Fuel and Electricity

**Figure 3:**
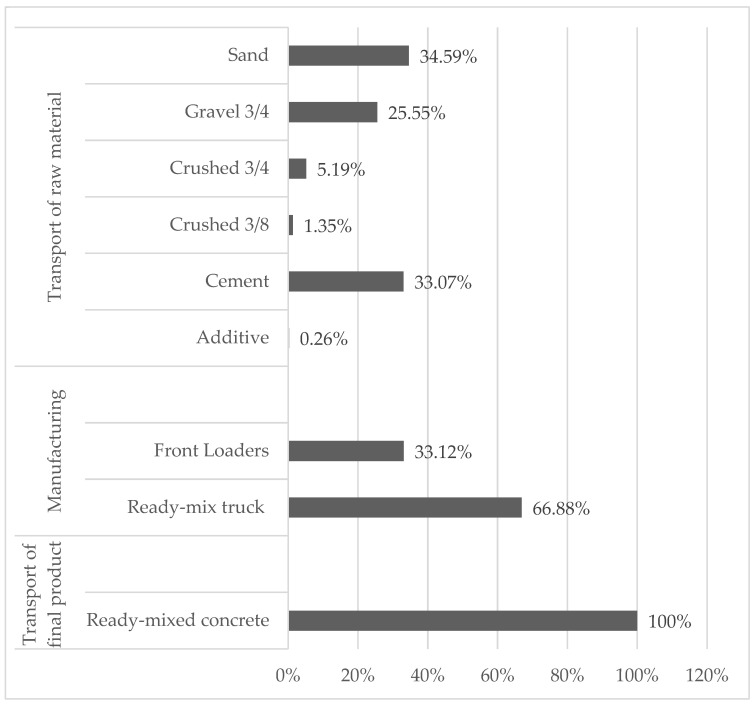
Annual fuel consumption by stages in relation to total consumption.

During the study year, a total of 1,354,236.24 L of diesel was consumed in the “transport of raw material” stage, 60,453.32 L of diesel in the direct transportation stage, and 264,539.19 L of diesel in the “transport of final product” stage to the construction site. The amount of fuel used within each stage in relation to the total is shown in Figure 3 and described below.

In the first stage, the highest fuel consumption was consumed in the sand and cement transport with 34.59% and 33.07%, respectively. Even though cement represents around 1800 dispatches in the year compared to 7300 dispatches of aggregates in general, cement consumes a representative amount of fuel. This is because the cement plant is in a different city (259 km) than the location of the ready-mixed concrete plant, which increases fuel consumption. It has been identified that in other countries the distances between the concrete plant and the cement plant are much shorter. For example, in the United States and Portugal, the distances are 112 km [46] and 60 km [39], respectively. Regarding the distance between the concrete plant and the aggregates quarries, the case study in Ecuador (around 50 km) and the study in Portugal (65 km) are below 100 km.

In the second stage, the front loader that transports the aggregates during the dosing process consumed 33.12% of fuel (diesel). The difference, 66.88% was used in the ready-mix trucks during concrete loading.

In the third stage, fuel consumed in the “transport of final product” represented a total route (round trip) of 308,826 km, (17,480 dispatches approximately).

Finally, the electricity consumption corresponding to the manufacture of ready mixed concrete reached 617,577 MJ. It represented 67.15% of the total electricity consumption of the plant (the difference corresponds to administrative activities).

### 3.2. Output Data

#### 3.2.1. Final Product

The annual production volume of ready-mixed concrete was quantified at 107,387 m^3^. Its production maintained a monthly fluctuation between 5.06% and 12.12% of the total volume produced in the year.

#### 3.2.2. Embodied Energy and CO_2_ Emissions

The total quantification of embodied energy and CO_2_ emissions in the ready-mixed concrete production within the established boundaries was considered. The values obtained were 61,069,813 MJ and 4,599,508 kg CO_2_, respectively. This value in the functional unit corresponds to 568.69 MJ/m^3^ and 42.83 kg CO_2_/m^3^ (Table 1).

**Figure 4:**
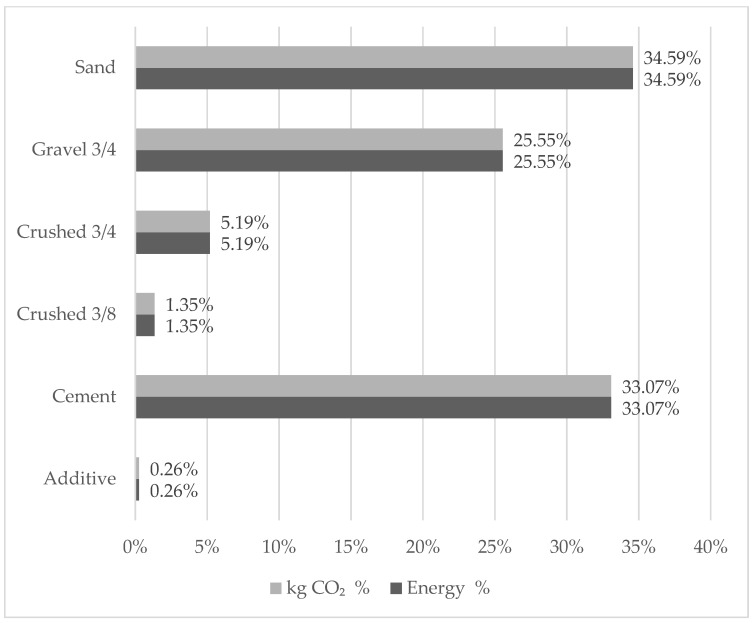
Embodied energy and CO_2_ emissions from the transport of each raw material.

Energy consumption and CO_2_ emissions quantified in the “transport of raw material” stage corresponds to 453.99 MJ/m^3^ and 33.67 kg CO_2_/m^3^, respectively. In this stage, the materials that required greatest amounts of fuel (sand and cement) are those that represents the highest incidence in the impact categories analyzed. Figure 4 shows the level of contributions between each material used.

**Figure 5:**
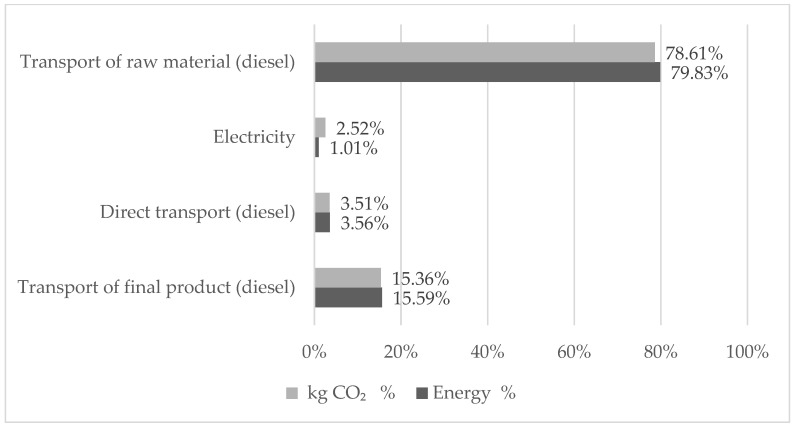
Embodied energy and CO_2_ emissions for each stage and type of energy consumed.

In the manufacturing stage, total energy consumption was 26.02 MJ/m^3^ (4.57%) and total CO_2_ emissions was 2.58 kg CO_2_/m^3^ (6.03%). There is a greater participation in energy consumption and CO_2_ emissions from transport compared to the electricity used (Figure 5). This coincides with data from countries in the region such as Chile or Mexico, which also reported energy consumption below 5% [14,64].

In relation to the last stage considered in the study, energy consumption for the “transport of final product” was 88.68 MJ/m^3^ (15.59%), which represents 6.58 kg CO_2_/m^3^ (15.36%).

Among the three stages, the first one corresponding to the “transport of raw material” presents the highest consumption of energy resources, with a percentage of 79.83% of the total energy consumption. This represents 78.61% of CO_2_ emissions. The next significant stage corresponds to the “transport of final product” and reaches 15.59% in energy consumption and 15.36% in CO_2_ emissions.

## 4. Discussion

This study in Ecuador shows that transport of raw materials has the greatest contribution to the environmental impacts of concrete, without considering cement. However, aggregates are also relevant in the overall environmental performance of concrete to avoid the scarcity of natural resources. In the analyzed ready-mixed concrete plant, it was found that the aggregates occupy 83.83% of the concrete components; however, alternative materials are not used to replace them. This is not uncommon since the research by [28] has identified that these studies are scarce in South America. Among the used recycled aggregates to replace virgin aggregates are construction and demolition waste [45]. A study in Ecuador analyzed the potential use of demolition concrete as an aggregate in concrete production and found that its application could be feasible in structural and non-structural elements [55]. Nevertheless, it has been identified that in one of the main local concrete plants, although they have begun to use recycled aggregates, the percentage is very low (1%) [56].

The ready-mixed concrete production flow diagram presented in this study consists of three stages. The first corresponds to the “transport of raw material” and represents 79.83% of the embodied energy and a carbon footprint of 78.61%. The second stage corresponding to manufacturing contributes 4.57% (diesel + electricity) in the embodied energy and corresponds to 6.03% of the carbon footprint. Finally, in the last stage of transporting the ready-mixed concrete to the work site, the embodied energy is 16.59% and represents 15.36% of the CO_2_ emissions. The first stage has the highest incidence in the defined categories of impact due to the following factors: distant suppliers, return conditions of transport units and high frequency. In the first and second cases, materials such as aggregates have distances that mostly exceed 50 km and cement that exceeds 285 km. However, considering the complete cycle of each trip (round trip), since the units return empty to their place of origin, the distances traveled and fuel consumption are double. In the third case, the high frequency of transfers of the materials required is due to the high demand for concrete production, which for the year of study reached 107,387 m^3^.

A study [47] carried out for Ecuador, confirmed that the raw material transport stage is the one that generates the most emissions in the manufacture of concrete after cement. This situation is similar to the results presented by [31], in which it was identified that cement and transport have the highest energy consumption.

Table 2 presents a compilation of scientific studies in which the embodied energy and CO_2_ emissions of different types of concrete are determined; among them, typical concretes, natural aggregates concrete (NAC), recycled aggregates concrete (RAC) and geopolymer concrete (GPC) are represented. Studies were also identified that quantify environmental impacts based on the compressive strength of concrete (between 18 MPa and 40 MPa) or simply refer to concrete in general [47]. Regarding the location of the studies, no type of restriction was considered because very few investigations quantify the embodied energy of the concrete production processes. Most studies focus on determining environmental impacts such as CO_2_. Regarding the system boundary of the analyzed studies, it was identified that mostly “cradle to gate” is used. However, there are also studies that use the “gate-to-gate” limit, such as the one presented in this study. In the first system boundary mentioned, it is possible to consider the stage corresponding to the transport of the final product to the construction site, while the second limit found can consider the stage corresponding to the transport of both the raw material and the construction site. From the information collected, it is indicated that the embodied energy values for the studies with a “cradle to gate” system boundary are between 1570.42 MJ/m^3^ and 5401 MJ/m^3^, while the CO_2_ values are between 225.84 kg CO_2_-Eq/m^3^ and 319.63 KgCO_2_-Eq/m^3^. It can be seen that there is not much variation between the studies that consider transportation to the construction site and those that do not, because this stage of transportation is not very representative in relation to the total [31]. On the contrary, if we analyze the “gate to gate” studies, the embodied energy values are between 55.95 MJ/m^3^ and 568.69 MJ/m^3^. Showing much larger differences than the previous limit. Likewise, it is identified that CO_2_ emissions are between 25.9 kg CO_2_-Eq/m^3^ and 256.78 KgCO_2_-Eq/m^3^. In this case, the difference in results is also significant. Some studies [14,45,65] already anticipated this situation and stated that it is mainly due to the following aspects: 1. Differences in the limits of the system, 2. Lack of details in the information on each process and sub-process, and 3. Specific conditions of the production processes.

In order to achieve a correct comparison between studies, it is necessary to delimit parts of the unitary processes to find similarity and reduce the asymmetry of the study cases. However, not all studies provide sufficient information. In this paper, three studies were compared. The first one in Latin America (Chile) and two with international databases: ECOINVENT and ICE. The first is a globally recognized database for its wide variety of life cycle inventory data for materials and production processes, while the second, ICE created at the University of Bath has data on concrete in general and on different pre-mixed concretes in relation to their mechanical resistance.

The research by [14], delimit the two international data-bases to compare their data with those obtained in the case of Chile. In the case of Ecuador, this information is used since the system limit is also from “gate to gate”. Thus, for each comparative case, particular parameters were defined (Table 3).

Case 1: Comparison between two Latin American countries. To compare the case of Ecuador with the results of the research from Chile [14], “transport of final product” was not considered. Stages: transport of raw materials + manufacturing.

Case 2: To compare with ECOINVENT data (SimaPro 7.3), the indirect transport was not considered. Stage: manufacturing.

Case 3: To compare with ICE version 2.0 data (January 2011), “transport of final product” was not considered. Stages: transport of raw materials + manufacturing.

In relation to the results presented in Table 3, in terms of embodied energy, the case study of Ecuador shows an increase of 40.27% compared to the case of Chile (Case 1), a decrease of 53.50% in respect to ECOINVENT data (Case 2) and 12.27% in respect to ICE database (Case 3). Regarding the case of Chile, one of the main aspects that can influence is the transport of raw materials. The data obtained by Ecuador is very high in relation to the average distances that are evidenced in the scientific literature (around 40 km) [45].

In terms of CO_2_ emissions, the results differ similarly in cases 2 and 3: the values corresponding to the case study of Ecuador are lower than baseline data from ECOINVENT and ICE in a percentage equivalent to 98.99% and 51.60%, respectively. While, in case 1, the annual value of CO_2_ emissions in the case study of Ecuador is 39.98% higher than the case study of Chile.

The high disparity in the results obtained in the comparison cases studies, in addition to what has already been mentioned, may be due to the diversity of fuels consumed in each process, since the energy matrices of each country are different. It also influences other aspects such as emissions factors, technologies and equipment used, therefore, the emissions associated with its energy production would also be different [14]. When comparing the results of a database suitability, especially for the European context, such as the case of ECOINVENT, with data from another region, significant differences may arise, such as those that are evident in the cases of Ecuador and Chile. An investigation determined that LCA studies still present many inconsistencies and uncertainties in the processes because very few studies detail each unit process [45]. This significantly hinders comparisons between investigations.

Additionally, most studies (70%) are based on secondary data and a small percentage use data specific to one location. The study of [45] showed that if generic data is replaced by collected data, variations can be found in the results of up to 20% in some indicators of environmental impact. This is because secondary data may not correctly reflect geography, time, and technology. In the same study, a review of various database sources showed that there is no database for the Latin American context.

Finally, there is a greater variation in the data when the system boundary is gate-to-gate than when it is cradle-to-gate. This is because the percentage of representation of cement production is really high. In the study of [31], it was determined that cement represents 79.94% of the total embodied energy, transport 17.01%, aggregates 1.77% and concrete 1.28%. This explains why there is a significant difference between concretes with different system limits.

## 5. Conclusions

Applying the LCA methodology according to ISO 14040, ISO 14044 and the 2006 IPCC Guidelines for National Greenhouse Gas Inventories, it was determined that in order to produce one cubic meter of ready-mixed concrete, 568.69 MJ of energy was required with an emission of 42.83 kg CO_2_. The system boundary considered is “gate to gate” including transportation (transport of raw materials and transport of final product).

The study determined that indirect transport, which corresponds to the transport used outside the system boundary (raw material and final product), represented 95.42% of the embodied energy and 93.97% of the CO_2_ emissions. However, the consumption of energy resources was mainly due to the transport of raw materials, since it represents 79.83%. In this same stage, the annual contributions of CO_2_ emissions to the environment represented 78.61%. Therefore, it is essential to consider alternatives to reduce these impacts. Perhaps, this can happen through the implantation of actions focused on reducing the number of dispatches of raw material, as well as looking for alternative sources of material exploitation to reduce the distances between the production plant and raw material suppliers.

Unlike previous studies, this research separately obtained the energy consumed and CO_2_ emissions of each stage of the life cycle in detail. For example, there is information on the transport of raw materials, quantification of raw materials, fuels used, and transportation of the final product to the construction site. Therefore, the results of this study can be easily used by other investigations.

On the other hand, it has been identified that the industry in Ecuador has great potential to reduce the embodied energy of its construction materials and, therefore, CO_2_ emissions. This is due to the fact that alternatives have not been sufficiently exploited to produce sustainable concrete and, thereby, also reduce the consumption of natural raw materials.

The high disparity between the results of studies that determine the embodied energy and CO_2_ emissions of concrete makes it difficult to make comparisons between case studies, due to the difference in system limits and the particular conditions established for each case study.

Due to the fact that in the country and particularly in the city of Cuenca, there are no databases of contained energy and CO_2_ emissions of construction materials, it is important to highlight the significant contribution of this research. Data on contained energy and CO_2_ emissions were provided for a specific case of ready-mixed concrete. A simple methodology was used to calculate these environmental impact categories, which can be replicated for the preparation of new studies that provide data on other construction materials. and thus have a national or regional database, the knowledge and application of which help to improve the manufacturing processes of construction materials with a focus on reducing environmental impact.

## Figures and Tables

**Table 1:** Embodied energy and CO_2_ emissions in each stage of the ready mixed concrete production.

Activities	Total Annual (MJ/m^3^)	Total Annual (kg CO_2_/m^3^)
Stage 1		
Transport of raw material	453.99	33.67
Stage 2		
Electricity	5.75	1.08
Direct transport	20.27	1.50
Stage 3		
Transport of final product	88.68	6.58
Total	568.69	42.83

**Table 2:** Comparison of embodied energy and CO_2_ emissions in concrete production between different studies and databases.

Study	Location	Concrete Specification	Embodied Energy	CO_2_ Emissions	System Boundary
[47]	Ecuador	Compressive Strength 18 MPa	-	225.84 kg CO_2_-Eq/m^3^	Cradle-to-gate
[47]	Ecuador	Compressive Strength 21 MPa	-	237.22 kg CO_2_-Eq/m^3^	Cradle-to-gate
[47]	Ecuador	Compressive Strength 24 MPa	-	256.08 kg CO_2_-Eq/m^3^	Cradle-to-gate
[47]	Ecuador	Compressive Strength 28 MPa	-	267.54 kg CO_2_-Eq/m^3^	Cradle-to-gate
[47]	Ecuador	Compressive Strength 40 MPa	-	355.38 kg CO_2_-Eq/m^3^	Cradle-to-gate
[66]	China	Geopolymer concrete (GPC) Compressive Strength 40 MPa	-	260.14 kg CO_2_/m^3^	Cradle-to-gate
[32]	Ireland	Typical concrete 30 MPa	1.08 MJ/Kg	-	Cradle-to-gate
[65]	Australia	Recycled concrete aggregate (RCA) Compressive Strength 20–40 MPa	4766–5401 MJ/m^3^	-	Cradle-to-gate **
SimaPro 7.3 ECOINVENT database [14]	50 countries, including Switzerland, France, Portugal and Sweden.	General concrete	55.95 MJ/m^3^	256.78 kg CO_2_/m^3^	Gate-to-gate
ICE 2.0 [14]	UK Britain	General concrete	547.2 MJ/m^3^	74.9 kg CO_2_/m^3^	Gate-to-gate *
[31]	Serbia	Natural aggregate concrete (NAC)	1570.42 MJ/m^3^	307.61 kg CO_2_-Eq/m^3^	Cradle-to-gate **
[31]	Serbia	Recycled aggregate concrete (RAC)	1613.02 MJ/m^3^	319.63 kg CO_2_-Eq/m^3^	Cradle-to-gate **
[14]	Chile	General concrete	342 MJ/m^3^	25.9 kg CO_2_/m^3^	Gate-to-gate *
This research	Ecuador	General concrete	568.69 MJ/m^3^	42.83 kg CO_2_/m^3^	Gate-to-gate *, **

* Including transportation of raw materials. ** Including transport to the construction site.

**Table 3:** Comparison of three concrete case studies in the “gate to gate” boundary system.

Comparison of Results		MJ/m^3^	kg CO_2_ /m^3^
Case 1	Case study Chile	342.2	25.9
	Case study Ecuador a	480.01	36.25
Case 2	SimaPro Studies 7.3 ECOINVENT database	55.95	256.78
	Case study Ecuador b	26.02	2.58
Case 3	ICE	547.2	74.9
	Case study Ecuador a	480.01	36.25

a Does not consider the stage “transport of final product”. b Does not consider the stage “transport of raw material” and “transport of final product”.

## Data Availability

Data are contained within the article.
